# Heritability and complex segregation analysis of deafness in Jack Russell Terriers

**DOI:** 10.1186/1746-6148-3-31

**Published:** 2007-11-13

**Authors:** Thomas R Famula, Edward J Cargill, George M Strain

**Affiliations:** 1 Department of Animal Science, University of California-Davis, Davis, CA 95616 USA; 2 Monsanto Company, 800 N Lindbergh Blvd, St Louis, MO 63167 USA; 3 Department of Comparative Biomedical Sciences, School of Veterinary Medicine, Louisiana State University, Baton Rouge, LA 70803 USA

## Abstract

**Background:**

The association between patterns of pigmentation and deafness in the dog has a long-documented history, with reports dating back over one hundred years. Long suspected of having a genetic basis, the search for loci with a pronounced influence in the expression of hearing loss in the dog has yet to be successful. No studies in the dog to date have found a possible influence of a specific colour locus associated with deafness. The present study is intended to evaluate the heritability of deafness in the Jack Russell Terrier (JRT), characterize the mode of inheritance, and evaluate the existence of a sex, coat colour, or coat texture influence on the expression of sensorineural deafness.

**Results:**

The estimation of heritability of deafness in the JRT was 0.22 when deafness was considered a binary (normal/deaf) trait and 0.31 when deafness was considered a three-category (normal/unilateral/bilateral deafness). The influence of coat colour in the incidence of JRT deafness was statistically significant, indicating that dogs with more white are more likely to be deaf. The influence of sex or coat texture was not statistically significant in the incidence of JRT deafness. Complex segregation analysis revealed a model of a single locus with a large effect on the binary measure of hearing loss is not supported.

**Conclusion:**

This is the first attempt, to our knowledge, to characterize a genetic component responsible for deafness in the JRT. The heritability of deafness in the JRT was found to be 0.22 and 0.31 considering deafness to be a two-category or three-category trait, respectively. There appears to be an influence of coat colour on the expression of deafness. In an attempt to characterize the mode of inheritance of deafness in the JRT, a model of a single locus with a large effect on hearing loss is not supported with this data. Further study is needed to determine if a single locus may be influencing deafness in the JRT. While the absence of a clear mode of inheritance complicates genetic dissection of deafness in the JRT, the assembling of this pedigree provides a tool for eventually defining the genetic bases of this disorder.

## Background

The association between patterns of pigmentation and deafness in the dog has a long-documented history, with reports dating back over one hundred years. Long suspected of having a genetic basis, the search for loci with a pronounced influence in the expression of hearing loss in the dog has yet to be successful. One study [1] has discussed various candidate loci based upon research on deafness in the dog, the human, and the mouse, but to date no specific loci have been shown to influence deafness in the dog. The interested reader is directed to a more comprehensive review of a molecular genetic approach to deafness in dogs, one outlining the application of comparative genomics [2].

Perhaps the most dramatic association between patterns of pigmentation and deafness in the dog can be found in the Dalmatian. In the Dalmatian, iris colour is positively correlated with deafness (dogs with at least one blue iris are more likely to be deaf) and the presence of a colour patch is negatively correlated with deafness (dogs with a colour patch are less likely to be deaf) [3-6]. No studies in the dog to date have found a possible influence of a specific colour locus (*i.e*., black or liver in the Dalmatian) associated with deafness [6].

There is a review of several breeds known to have a high risk for pigment-associated sensorineural deafness [6]. In addition to the Dalmatian, the review reported evidence showing white Bull Terriers are more likely to be deaf than Bull Terriers with colour in their coats. The Jack Russell Terrier (JRT) was also examined, however with records on only 56 dogs it could only be noted that 47 of the 56 dogs had normal hearing, with 4 dogs being unilaterally deaf and 5 bilaterally deaf. More observations would be needed in order to attempt to substantiate a genetic component to hearing loss in the JRT.

**Figure 1:**
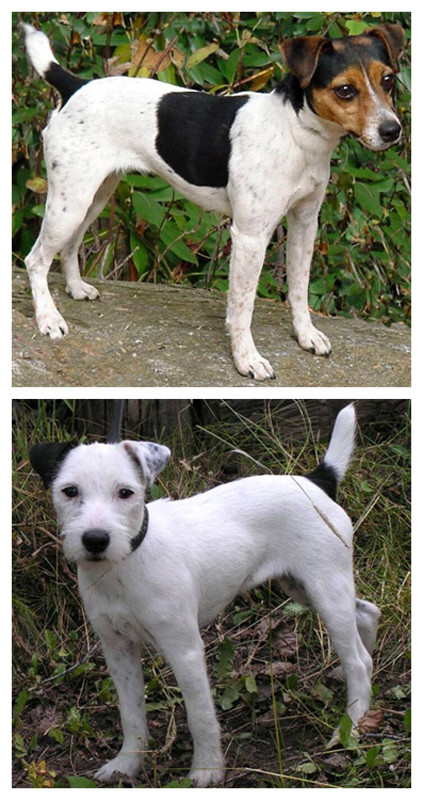
**Illustration of Jack Russell Terrier phenotypic variation**. (Top) A tri-colour (brown, black, white) and smooth coat texture JRT. Notice the areas of white are not pure white as specks of black can be seen in the white on the hindquarter, forequarter, and main body. (Bottom) A black and white, broken coat texture JRT. Notice the overall amount of white present compared with the JRT pictured in the top. Minimal specks of black can be seen in the forequarter and the dog's left ear, but the white on the main body and hindquarter has little to no specks of colour. This dog has a broken coat texture; a combination of rough and smooth, with the rough texture on its nose/head and the majority of the rest of its coat is smooth. These dogs were not included in the dataset presented in this study and their images are included for illustrative purposes only.

The breed standard for the JRT includes a requirement that the dog's body be more than 51% white with tan, black, or brown markings [7]. The requirement that a JRT coat be 51% or more white creates variation within the breed ranging from dogs with a large portion of colour in their coats (up to 49%) to dogs that are mostly white (*i.e*., 90% or more white) as shown in Figure 1. The JRT standard also describes three different types of coat texture: smooth, rough, and broken (a combination of smooth and rough) [8]. Examples of smooth texture and broken texture are shown in Figure 1. The JRT can also have blue eyes, but a JRT with blue eyes is rare compared to the incidence in other breeds (*e.g*., the Dalmatian). To the author's knowledge, no previous study has examined the influence of coat colour or coat texture on deafness in the JRT.

The present study is intended to evaluate the heritability of deafness in the JRT, characterize the mode of inheritance, and evaluate the existence of a sex, coat colour, or coat texture influence on the expression of sensorineural deafness.

## Results

**Figure 2:**
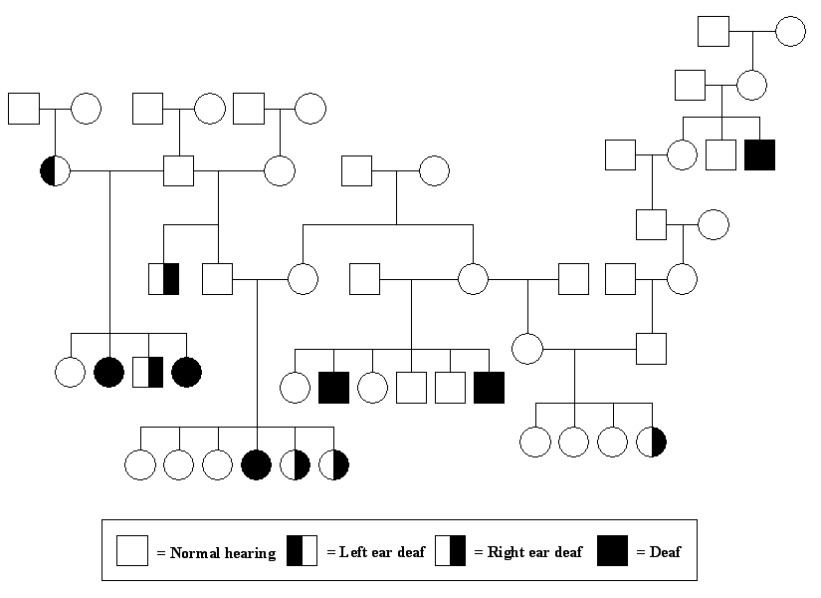
Subset of 51 Jack Russell Terriers with known auditory status from the assembled pedigree.

Of the 201 dogs (94 males, 107 females) with known auditory status, 176 dogs (87.6%) had normal hearing, 16 (7.9%) were unilaterally deaf and 9 (4.5%) were bilaterally deaf (Table 1). Figure 2 shows a subset pedigree of 51 dogs with known auditory status from the dataset. There are several males with a great impact on this pedigree (data not shown), including a few instances of inbreeding. Specifically, of the 236 total animals represented in the pedigree, 12 were inbred, with an average inbreeding coefficient of 0.055 as calculated using the program MTGSAM [9]. A preliminary analysis of the deafness data without considering the potential contribution of inheritance (i.e. using a logit analysis in the R program [10] with effects of sex, coat colour and coat texture) found none of the included fixed effects to be significant contributors to the incidence of deafness (results not shown). Similar conclusions (i.e., no significant contributions from sex, coat colour or coat texture) could be drawn from a preliminary analysis of the three-category phenotype of deafness using a multinomial model in R[10].

**Table 1:** Recorded phenotypes^a ^of Jack Russell Terriers.

Phenotype	Female	Male
Hearing/Smooth/White	2	4
Hearing/Rough/White	7	5
Hearing/Broken/White	2	4
Hearing/Smooth/Tricolour	7	5
Hearing/Rough/Tricolour	6	8
Hearing/Broken/Tricolour	5	4
Hearing/Smooth/Tan	5	5
Hearing/Rough/Tan	18	5
Hearing/Broken/Tan	8	12
Hearing/Smooth/Black	2	4
Hearing/Rough/Black	0	1
Hearing/Broken/Black	3	2
Unilateral/Smooth/White	0	0
Unilateral/Rough/White	2	1
Unilateral/Broken/White	2	0
Unilateral/Smooth/Tricolour	2	0
Unilateral/Rough/Tricolour	1	1
Unilateral/Broken/Tricolour	0	1
Unilateral/Smooth/Tan	1	0
Unilateral/Rough/Tan	1	2
Unilateral/Broken/Tan	0	0
Unilateral/Smooth/Black	0	0
Unilateral/Rough/Black	0	0
Unilateral/Broken/Black	0	0
Unilateral/Smooth/White	0	0
Deaf/Smooth/White	0	1
Deaf/Rough/White	1	1
Deaf/Broken/White	0	1
Deaf/Smooth/Tricolour	0	0
Deaf/Rough/Tricolour	0	2
Deaf/Broken/Tricolour	0	0
Deaf/Smooth/Tan	1	0
Deaf/Rough/Tan	1	0
Deaf/Broken/Tan	0	1
Deaf/Smooth/Black	0	0
Deaf/Rough/Black	0	0
Deaf/Broken/Black	0	0

^a ^Auditory status/coat texture/coat colour and sex.

^b ^Phenotypes of Jack Russell Terriers from the assembled pedigree. Dogs with at least one unknown trait are not included in this table, but were included in the dataset.

Table 2 offers our first examination of the role inheritance may play in deafness in the JRT. Whether considered a trichotomous trait (three categories of response) or a dichotomous trait (two categories of response), both analyses presented in Table 2 suggest that deafness has an appreciable genetic component. For example, the set of Gibbs samples considered in Table 2 reveal that the median value for the heritability of dichotomous deafness is 0.22 and 0.31 for trichotomous deafness (on the underlying continuous scale). Though derived from a relatively small sample of JRT, the heritability estimates suggest selection to reduce the incidence of this disorder should be appreciable within several generations.

**Table 2:** Estimation of heritability^a^

	Mean	Median	SD	Effective Sample Size	Convergence Score (p-value)^b^	95% HDR^c^	Relative Risk^d^
*Dichotomous Trait*							
Genetic Variance	0.44	0.28	0.55	1664.7	0.90 (0.37)	0.06,1.83	
Heritability	0.26	0.22	0.16	1746.4	0.94 (0.35)	0.06,0.65	
White – Tricolour	0.69	0.68	0.42	8930.9	1.39 (0.16)	-0.12,1.51	1.51
White – Tan	0.83	0.82	0.40	8384.9	-0.74(0.46)	0.06,1.62	1.58
Female-Male	-0.23	-0.22	0.30	8619.0	0.13 (0.90)	-0.82,0.36	0.95
*Trichotomous Trait*							
Genetic Variance	0.80	0.45	1.29	1681.2	1.07 (0.28)	0.12,3.67	
Heritability	0.35	0.31	0.18	1786.5	1.10 (0.27)	0.11,0.79	
White – Tricolour	0.74	0.74	0.40	9868.5	1.19 (0.23)	-0.05,1.54	1.64
White – Tan	0.84	0.84	0.39	8535.9	0.62 (0.53)	0.06,1.61	1.63
Female-Male	-0.31	-0.31	0.29	9496.6	-0.18 (0.85)	-0.89,0.25	0.93

^a ^Estimates are taken from a Gibbs sample of 10,000 values.

^b ^Gibbs sample convergence statistic; Geweke (1992).

^c ^High Density Region.

^d ^Relative risk is computed for the probability of at least one deaf ear for white male versus tricolour male, white male versus tan male and white female versus white male, respectively.

Also presented in Table 2 is the influence of coat colour in the incidence of JRT deafness. Consistent for each measure of hearing loss, the difference between mostly white dogs and those with a tan colour pattern indicates that dogs with more white are more likely to be deaf. This conclusion is justified by examination of the empirical 95% Highest Density Region (HDR) generated by the 10,000 Gibbs samples. Specifically, the comparison of white and tan coloured dogs found that 95% of the Gibbs sample estimates of this difference are between 0.06 and 1.61 (on the underlying unobservable continuous scale), an interval that does not overlap with 0. Given that we scored the trait 1 for deaf and 0 for unaffected, larger values of the parameter θ (from equation [e1]) imply a greater risk for deafness. Only the contrast for white and tan coated dogs had an HDR that did not overlap 0. Accordingly, only selected contrasts are presented in Table 2. As mentioned previously, there was only one black and white JRT with deafness (unilateral) in the pedigree, hence there was insufficient data to analyze this separate coat colour category and its possible influence on deafness. However, the contrasting results observed between the mostly white dogs and those with a tan colour pattern are sufficient to conclude a colour influence on the expression of deafness in the JRT. The contrast for coat texture had an HDR overlapping 0 (data not shown). Note, the significant contribution of the tan coat colour stands in contrast to the result found when the genetic contribution to deafness was not considered.

**Figure 3:**
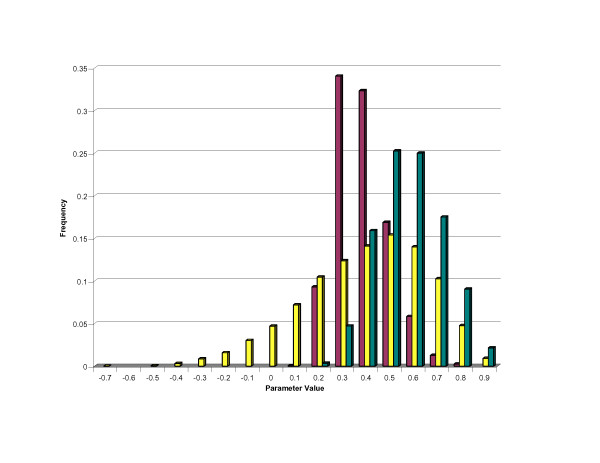
Frequency of estimated genetic parameter values from the Gibbs sample for the heritability of deafness , the heritability of coat colour  and the genetic correlation between deafness and coat colour .

Table 3 presents results of the multiple trait evaluation of deafness with the binary coat colour score, where deafness is scored as both a dichotomous and a trichotomous trait. The estimates of heritability for deafness are roughly equivalent to those for the univariate analyses in Table 2. Similarly, there is no evidence in the multiple trait analysis for gender differences in the risk of hearing loss. As one might expect, the heritability of our binary coat colour score appears to be moderately high. In addition, sex of the dog does not seem to be related to the presence or absence of colour in a dog's coat. As in the univariate analyses, coat texture did not have a significant influence on deafness or the binary coat colour score. The estimate of the genetic and phenotypic correlations between deafness and coat colour are not significantly different from zero, as evidenced by the 95% HDR and its overlap with zero. A graphical representation of the results of this analysis can be found in Figure 3. Specifically we see the frequency of values for the heritability of deafness, the heritability of coat colour and the genetic correlation between these characters on the underlying scale taken from the 10,000 Gibbs samples. Accordingly, one can clearly visualize the considerable overlap of the genetic correlation on both sides of the central value of zero (0.0).

**Table 3:** Estimation of heritability and genetic correlation^a^

	Mean	Median	SD	Effective Sample Size	Convergence Score (p-value)^b^	95% HDR^c^
*Dichotomous Deafness Trait*						
Genetic Variance	0.54	0.46	0.31	2291.9	0.98 (0.33)	0.13,1.11
Heritability	0.33	0.32	0.11	2209.9	1.51 (0.13)	0.14,0.54
Female-Male	-0.23	-0.22	0.29	9198.2	0.75 (0.45)	-0.79,0.33
*Binary Coat Colour Trait*						
Genetic Variance	1.32	1.06	0.94	1206.3	1.68 (0.09)	0.20,3.14
Heritability	0.52	0.51	0.14	1380.9	1.52 (0.13)	0.26,0.79
Female-Male	-0.26	-0.25	0.35	8497.4	-0.29 (0.77)	-0.95,0.40
*Correlation of Dichotomous Deafness and Binary Coat Colour*						
r_g_^d^	0.34	0.36	0.25	3258.1	1.55 (0.12)	-0.15,0.79
r_p_^e^	0.22	0.23	0.12	4313.9	1.40 (0.16)	-0.10,0.47
*Trichotomous Deafness Trait*						
Genetic Variance	0.93	0.78	0.56	1408.8	0.28 (0.78)	0.23,1.94
Heritability	0.45	0.44	0.12	1749.8	0.23 (0.81)	0.23,0.68
Female-Male	-0.33	-0.33	0.31	9379.2	0.65 (0.52)	-0.93,0.29
*Binary Coat Colour Trait*						
Genetic Variance	1.39	1.09	1.12	922.0	-0.74 (0.46)	0.18,3.37
Heritability	0.53	0.52	0.14	1052.4	-0.45 (0.65)	0.26,0.80
Female-Male	-0.26	-0.25	0.36	8427.6	1.17 (0.24)	-0.98,0.44
*Correlation of Trichotomous Deafness and Binary Coat Colour*						
r_g_	0.34	0.36	0.24	2907.3	0.10 (0.92)	-0.12,0.76
r_p_	0.25	0.25	0.13	3036.1	-0.01 (0.99)	-0.01,0.50

^a ^Estimates are taken from a Gibbs sample of 10,000 values.

^b ^Gibbs sample convergence statistic; Geweke (1992).

^c ^High Density Region.

^d ^Genetic correlation.

^e ^Phenotypic correlation

Table 4 presents results of the complex segregation analysis of dichotomous deafness. However, further discussion of these results is not necessary, given that a model of a single locus with a large effect on our binary measure of hearing loss is not supported. This conclusion is based upon the 95% HDR shown for both the general major locus model and the completely recessive major locus model in Table 4, where the estimate of the variance attributable to the putative major locus includes zero (0.0). A second demonstration of the failure of each of these major locus models to provide an adequate explanation of the patterns of inheritance of deafness is that the 95% HDR for the allele frequency includes the value 1.0. Taken together, these results show that the no major locus model provides a plausible explanation for the inheritance of deafness in the JRT. Note, however, that our evaluation of a major locus may be influenced by possible ascertainment bias, our sample of dogs being built upon affected animals. Because ascertainment bias raises the probability of a false positive declaration of the presence of a major locus, it would appear its impact here was not influential.

**Table 4:** Mixed-inheritance model parameters^a ^for dichotomous deafness in Jack Russell Terriers

	Polygenic Variance	Major Locus Variance	Additive Effect (a)	Dominance Deviation (d)	τ_AA_^b^	τ_AB_	τ_BB_	Frequency (q)
General Major Locus, Mendelian Transmission								
Mean	1.48	8.46	3.64	-2.65	1.0	0.5	0.0	0.84
Median	1.53	9.72	3.89	-2.57	-	-	-	0.83
SD	0.84	10.21	1.18	1.35	-	-	-	0.11
Eff Sample Size^c^	1417.3	1007.3	980.2	1013.6	-	-	-	1198.6
Conv Score (p)^d^	1.32 (0.19)	1.55 (0.12)	1.68 (0.09)	1.71 (0.09)	-	-	-	1.49 (0.14)
HDR^e ^95% Low	0.00	0.00	1.48	-5.21	-	-	-	0.38
HDR 95% High	3.22	33.14	7.77	0.06	-	-	-	1.00
Recessive Major Model, Mendelian Transmission								
Mean	2.21	13.19	3.68	-3.68	1.0	0.5	0.0	0.74
Median	2.33	12.32	3.65	-3.65	-	-	-	0.75
SD	0.60	6.03	0.84	0.84	-	-	-	0.41
Eff Sample Size	1330.9	1121.0	1032.6	1032.6	-	-	-	1064.1
Conv Score (p)	-1.27 (0.20)	1.51 (0.13)	1.74 (0.08)	1.74 (0.08)	-	-	-	1.42 (0.16)
HDR 95% Low	0.39	0.00	2.15	-6.23	-	-	-	0.44
HDR 95% High	3.16	45.89	7.42	-1.03	-	-	-	1.00
General Major Locus, Non-Mendelian Transmission								
Mean	1.46	6.53	4.13	-3.01	0.63	0.52	0.18	0.94
Median	1.57	6.34	4.36	-3.29	0.70	0.51	0.01	0.99
SD	0.88	8.18	1.62	1.57	0.05	0.05	0.13	0.03
Eff Sample Size	1387.6	1109.2	1290.3	1088.1	1172	1043	941	1186.4
Conv Score (p)	1.18 (0.24)	1.40 (0.16)	1.37 (0.17)	1.19 (0.23)	1.08 (.28)	1.19 (.23)	1.75 (.08)	0.97 (0.33)
HDR 95% Low	0.00	0.00	0.53	-7.11	0.52	0.26	0.00	0.75
HDR 95% High	3.23	33.69	8.04	-0.48	0.86	0.66	0.63	1.00

^a ^Estimates are taken from a Gibbs sample of 9,000 values.

^b ^Mendelian transmission parameter; the probability of transmitting an "A" allele. For Mendelian transmission these values are fixed as 1.0, .50 and 0.0 for putative major genotypes AA, AB and BB, respectively. Non-Mendelian transmission implies estimation of these values from the data.

^c ^Effective sample size.

^d ^Gibbs sample convergence statistic; Geweke (1992).

^e ^Highest density region

Not presented is an analysis of the trichotomous definition of deafness because such a phenotype cannot be evaluated legitimately with the iBay software; the software is limited to the evaluation of binary and normally distributed phenotypes. However, fitting the values of normal hearing, unilateral deafness and bilateral deafness as scores of 0, 1, 2, respectively, did support the conclusion of a segregating major locus (through examination of the 95% HDR).

## Discussion

This is the first attempt, to our knowledge, to characterize a genetic component responsible for deafness in the JRT. It is clear from the results presented in Table 2 that deafness in the JRT is hereditary and is influenced by genetic information passed from parent to offspring. As such, the heritability of deafness is of sufficient magnitude that attempts to select against it are potentially successful.

However, the heritability of deafness reported here for the JRT is lower than the estimates of the heritability of deafness in the Dalmatian [3-5], the only other breed with such estimates to the authors' knowledge. While the prevalence of deafness in these respective breeds is also not the same [6], as the JRT is less affected compared to Dalmatians, differing estimates of the heritability of deafness between the breeds could be suggestive of dissimilar mechanisms resulting in a similar phenotype. There are obvious issues (*e.g*., pedigree structure, pedigree size, *etc*) in comparisons between studies analyzing estimations of heritability within one breed. Those issues also apply in comparison of this study to any conducted utilizing another breed. However, since this is the only study to date analyzing deafness in the JRT, comparisons to studies in the Dalmatian are all that are available.

Unlike the Dalmatian, where the heritability of deafness has been estimated to be as high as 0.75 for a trichotomous trait [3], the highest heritability estimate in the JRT in this study of 0.31 for a trichotomous trait is not indicative by itself of a single major locus exerting a large effect. It has been demonstrated [11] that major loci tend to increase the heritability of a trait in a given population and a value greater than 0.70 is comparatively large for many polygenic traits. Because the estimate in the JRT is markedly below 0.70, other loci may be exerting an effect on any major locus responsible for deafness in the JRT. To further support this, Table 4 presents the results of the complex segregation analysis whereby the model of a single locus with a large effect on hearing loss is not supported.

Though we can conclude that deafness is heritable from the results in Table 2, the exact genetic mechanism that leads to expression of this disease cannot be stated with certainty based on the results in Table 4. A manual review of the pattern of inheritance did not support a model of a simple autosomal Mendelian locus. For example, the majority of the affected progeny were the result of matings of two unaffected parents, eliminating models of a single dominant deafness allele. Discarding a model of a single recessive autosomal allele is not possible with the pedigree, because there were no matings of two bilaterally deaf dogs; nor was there any mating of two unilaterally deaf dogs. Once again, the reader is reminded that our evaluation of a major locus may be influenced by possible ascertainment bias, our sample of dogs being built upon affected animals. Regrettably, there is no simple means by which this potential effect can be eliminated from the present analysis. What remains is the need for a cautious interpretation of the results of the several analyses.

Also presented in Table 2 is the influence of coat colour and coat texture in the incidence of JRT deafness. Consistent for each measure of hearing loss, there is a difference between mostly white dogs and those with a tan colour pattern. This indicates that mostly white dogs are more likely to be deaf, supporting that deafness in the JRT (as in other breeds such as the Dalmatian); there is a pigmentation association with deafness. The breed standard for the JRT mandates that the body be at least 51% white [7]. Unfortunately, no JRTs with blue eyes were available with this pedigree to evaluate a possible association between eye colour and deafness as has been observed in the Dalmatian. However, as the multiple trait analysis presented in Table 3 reveals, the association between coat colour and deafness appears to be less pronounced in the JRT than in the Dalmatian. Though the mean estimate of the genetic correlation (taken as the mean of the Gibbs sample) is strong and positive (e.g., a value of 0.34 for dichotomous deafness with the binary coat colour score), the 95% HDR suggests that estimate to be quite imprecise. Perhaps a larger sample of dogs, or a better means of quantifying coat colour would reveal a more precise relationship between hearing loss and pigmentation.

Although deafness in the JRT is clearly inherited, the evidence for the presence of a single major gene affecting the disorder is not persuasive with the data from this pedigree. In a review of complex segregation analysis [12], it was suggested to exercise caution in the interpretation of complex segregation analysis until several sets of data had confirmed or rejected the presence of a Mendelian locus. Further studies will be valuable in this context.

## Conclusion

The objectives of this study were to evaluate the heritability of deafness and the existence of a sex, coat colour, and/or coat texture influence on the expression of deafness in the JRT as well as characterize the mode of inheritance. The heritability of deafness in the JRT was found to be 0.22 and 0.31 considering deafness to be dichotomous and trichotomous, respectively. There appears to be an influence of coat colour on the expression of deafness as a difference was observed between mostly white JRTs and those with a tan colour pattern, indicating that white JRTs are more likely to be deaf. In an attempt to characterize the mode of inheritance of deafness in the JRT, a model of a single locus with a large effect on hearing loss is not supported with this data. Further study is needed to determine if a single locus may be influencing deafness in the JRT.

While the absence of a clear mode of inheritance complicates genetic dissection of deafness in the JRT, the assembling of this pedigree provides a tool for eventually defining the genetic bases of this disorder. Initially, one study [13] reported a heritability estimate of 0.32 in Californian Dalmatians, a value comparable to the estimate presented here in JRTs. However, a subsequent study [4] included more records of Californian Dalmatians in a larger data set and reported a higher heritability estimate of 0.76, a value comparable to the estimates presented by other studies involving Dalmatians [3,5]. Inclusion of more individuals related to those in this pedigree may provide the opportunity for a more in-depth analysis of the heritability of deafness in JRTs. Also, collection of additional unilaterally or bilaterally deaf JRTs with black and white coat, as well as collection of normal and affected JRTs with blue eyes, will further elucidate the influence of pigmentation on the expression of deafness in this breed.

## Methods

### Phenotypic data

Phenotypes for hearing loss were measured using the brainstem auditory evoked response (BAER), permitting the discrimination between normal hearing dogs and bilaterally or unilaterally deaf dogs. Data were collected on 236 dogs from one large family, starting with a deaf proband and her immediate relatives, then extending broadly to available relatives. The majority of animals originated largely from the eastern United States (US), but subjects are included from across the country. BAER testing was typically done in puppies at five weeks of age, but several were tested as adults. Average test age is not known. Out of the 236 dogs, 201 have a known auditory status. The additional 35 dogs were included to help build appropriate pedigrees despite having an unknown auditory status. BAER measurements were previously performed at various test sites in the US at the owners' initiative. Copies of BAER test results were collected as confirmation of each animal's hearing status. BAER testing followed standard methods [6]. Figure 2 illustrates a subset of 51 dogs with known auditory status from this pedigree. Phenotypic information on sex, coat colour, coat texture, auditory status, and eye colour were collected by use of a standardized form distributed and returned by mail. In addition, pedigree information was recorded, along with the BAER results and phenotypic information of relatives where known. Colour classes were defined as white, tri-coloured, tan, or black, and coat texture was described as smooth, rough or broken [8]. There were no JRTs in the assembled pedigree with blue eyes. Also of note, only one dog with black and white coat colour was collected; a dog also affected by deafness (unilateral). The remaining JRTs with black and white coat colour in the pedigree have normal hearing or unknown auditory status.

An initial evaluation of the data was conducted with the R-program [10] without consideration of the pedigree or relationships among dogs in the data set. The purpose was to evaluate the potential impact of fixed effects such as sex or coat colour on deafness without regard to the genetic contributions to this disease. Analysis of deafness as a binary trait included models with effects for sex, coat colour and coat texture (along with all possible interactions) using a logit link function under the "glm" command of the R-program[10].

### Estimation of heritability

The BAER, used to determine the auditory function of each ear, provides for two possible deafness phenotypes in this pedigree. One phenotype is dichotomous, in which unilaterally deaf and bilaterally deaf dogs are classified as deaf (*i.e*., affected vs. unaffected). An alternative phenotype is trichotomous, with classes for normal hearing, unilateral deafness and bilateral deafness, representing ordered categories of increasing disease.

Most data sets utilized in the study of hereditary diseases are constructed around probands, making correction for ascertainment bias necessary; this set of data is no exception. In estimation of heritability, mixed linear models are capable of accommodating non-randomly sampled data [14]. Accordingly, the estimation of the heritability of deafness should not be biased by family selection, provided that the animals at the top of the pedigree (those animals with no parents identified) can be considered a random sample of JRTs. This is more assumption than assertion because it is not feasible to create or discount a process of selection against deafness or for sampling such animals disproportionately among those animals at the top that have no known auditory status.

We choose to estimate heritability through the use of threshold models [15], an approach typical for the analysis of binary and ordered categorical traits. For example, we consider the phenotype of deafness as a binary trait y_ijkl _(where y_ijkl _= 0 when unaffected; 1 when affected) for the *l*-th dog (*l *= 1, 2,...201) of the *i*-th sex (*i *= 1 for males; 2 for females) in the *j*-th coat texture class (*j *= 1 for smooth; 2 for rough; 3 for broken) and the *k*-th coat colour class (*k *= 1 for white; 2 for tricolour; 3 for tan; 4 for black). In threshold models, this binary phenotype is assumed to be related to an underlying, unobservable, normally distributed continuous variable, θ, through a set of three fixed thresholds, [γ_0 _= -∞; γ_1 _= 0; γ_2 _= ∞]; γ_1 _is set to zero for computational convenience, with no loss in generality or impact on subsequent analysis. Accordingly, the combination of continuous genetic and environmental terms thought to control the unobservable θ are translated into a categorical observation through comparison to the fixed thresholds (*i.e*., observe an unaffected dog when γ_0 _≤ θ < γ_1 _or an affected dog when γ_1 _≤ θ < γ_2_).

We also consider deafness as a trichotomous trait, in which normal hearing dogs are scored as a zero, unilaterally deaf dogs scored as a one, and bilaterally deaf dogs are scored as a two. Such a characterization of the auditory phenotype requires only minor modification of the threshold model. Specifically we add a fourth fixed threshold [γ_0 _= -∞; γ_1 _= 0; γ_2_; γ_3 _= ∞], where γ_2 _must be estimated from the data. Furthermore, normal hearing dogs would be observed when γ_0 _≤ θ < γ_1_, unilaterally deaf dogs would be observed when γ_1 _≤ θ < γ_2_, and bilaterally deaf dogs would be observed when γ_2 _≤ θ < γ_3_.

The model for θ is similar to any that can be used for continuous phenotypes. The algebraic form of the model for this study is:

θ_ijkl _= μ + sex_i _+ texture_j _+ colour_k _+ a_l _+ e_ijkl _

where θ_ijkl _is an unobservable continuous variate for the *l*-th (*l *= 1, 2,..., 201) dog of the *i*-th sex in the *j*-th class of coat texture and the *k*-th coat colour class. The component μ is an unknown constant while sex_i _is the contribution of the *i*-th sex to the expression of deafness. Coat texture_j _and coat colour_k _are similar contributions of these physical characteristics to the liability for deafness; a_l _is the additive genetic contribution of the *l*-th animal and e_ijkl _is an unknown residual. Both a_l _and e_ijkl _are assumed to be random effects with zero means and variances of σ_a_^2 ^(the additive genetic variance) and σ_e_^2 ^(the residual variance), respectively. The additive genetic effect for each animal accounts for the covariance in phenotypes of relatives and is assumed to be multivariately-normally distributed, with a covariance structure based upon the additive relationships among all 236 animals. Because the underlying scale is unobservable, the total variance is assumed to be σ_P_^2 ^= σ_a_^2 ^+ σ_e_^2 ^where σ_e_^2 ^= 1.0, with no loss of generality [16-18]. The heritability of deafness, on the unobservable continuous scale, can be estimated as h^2 ^= σ_a_^2^/(σ_a_^2 ^+ σ_e_^2^).

To estimate the unknown fixed effects and unknown σ_a_^2 ^we used a mixed model Bayesian strategy [18]. An advantage of Bayesian methods is the ability to arrive at not only a point estimate of the unknown parameters (*e.g*., heritability), but also a distributional estimate. Though a more complete description of the statistical aspects of this analysis is available [17], briefly, the assumed prior densities for the fixed effects (sex, coat texture and colour effects) are uninformed, what Bayesian modellers refer to as a "flat" prior density. That is, we assume no prior knowledge of the magnitude of the fixed effects, allowing for the possibility that any value along the real line is a possible value. For the analysis of deafness as a binary observation there is no need to estimate the fixed thresholds. However, for the case of the trichotomous deafness, γ_2 _must be estimated. The assumed prior distribution for this parameter is the uniform with bounds established by γ_1 _and γ_3_. As for the random contributions to θ, the additive genetic effects are assumed to be multivariately-normally distributed with a null mean and variance-covariance structure consisting of the numerator relationship matrix times the unknown additive genetic variance, σ_a_^2^. Similarly the random residuals are assumed to be independently normally distributed with null mean with variance σ_e_^2 ^= 1.0 (with no loss of generality since θ is an unobservable variate). Finally, given our Bayesian approach to this problem, we also must establish a prior density for the unknown variance σ_a_^2^. Specifically, we look to the inverted Wishart distribution where the expected prior mean for the additive genetic variance was started at 1.0, with a shape parameter of 3. The shape parameter reflects the degree of certainty we have in the choice of the prior mean for the additive genetic variance (the larger the value the more certain). A value of 3, speaking relatively, would be considered small, reflecting weak prior knowledge of the actual value for the additive genetic variance.

Estimation of the distribution of the unknown parameters employs a technique of numerical integration referred to as Gibbs sampling [19]. The algorithm is based on the iterative generation of a sequence of random variables from the known conditional distributions of the parameters, given the likelihood function of the data. Subsequent estimates of the parameters are found in the analysis of this sequence of random numbers, called the Gibbs sample. A more complete description of the Gibbs sampling process and its theoretical justification is available [18], as well as in the manual of the public domain software, MTGSAM [9], with which this analysis was performed.

In this study, the total length for the Gibbs sampling process was set to 300,000, with the first 50,000 samples discarded from any subsequent analysis (called the "burn-in"). The post-Gibbs analysis was implemented with the packages boa [20] and coda [21], both part of the R-program [10]. Convergence of the Gibbs sampling process was evaluated visually by sample plots and by a diagnostic test contrasting sample means from the first 10% of the sample with the last 50% of the sample [22]. Autocorrelations were calculated within the complete Gibbs sample to arrive at a suggested thinning rate. Gibbs sample statistics, including effective sample size, were calculated with a thinning rate of 25 (chosen based on computation of a maximum autocorrelation at lag 25 of 0.02 for all parameters), creating a final Gibbs sample of 10,000 sample observations (*i.e*., [300,000-50,000]/25 = 10,000). Highest density regions (HDR) were computed as described [23] with public domain software hdrcde, [24] a package within the R-program [10].

To further assess the relationship between deafness and coat colour, we also considered a multiple trait analysis of these two categorical phenotypes. Such an analysis permits estimation of the genetic correlation between hearing loss and coat colour on the underlying unobservable scale. The challenge of such an analysis is in finding the appropriate means to score coat colour. Because there is no way to rank coat colour differences on a presumed scale of colour, we chose a binary definition of coat colour, where dogs classified as white were given a score of 1.0 and all other colour combinations (e.g., tri colour, tan) were given a score of zero (0.0).

With this binary score for coat colour, we could then use MTGSAM [9] to estimate the genetic correlation of the two traits (along with estimates of heritability fro deafness and for this binary scoring of coat colour). The algebraic form for the underlying unobservable variate would then take the form:

θ_ijkl _= μ + sex_il _+ texture_jl _+ a_kl _+ e_ijkl _

where θ_ijkl _is an unobservable continuous variate for the *k*-th (*k *= 1, 2,..., 201) dog of the *i*-th sex in the *j*-th class of coat texture for the *l*-th trait (*l *= 1 for deafness and *l *= 2 for binary coat colour). The terms for sex, coat texture and animal effect are as described in model [e1]. This extension of model [e1] requires estimation of two genetic variances, one for deafness and the second for coat colour, a genetic covariance between these two categorical phenotypes and a residual covariance between the two phenotypes. As described above for the univariate deafness model, unknown parameters were estimated from a Gibbs sample of 10,000 values.

### Complex segregation analysis

The possibility that deafness in JRTs is influenced by the action of a segregating locus of large effect can also be examined. This technique, called complex segregation analysis [25], is intended to integrate Mendelian transmission genetics and models of penetrance with the patterns of covariance expected in polygenic inheritance. A more complete description of complex segregation analysis is available [26].

An outline of the criteria that must be satisfied before acceptance of the single major locus model has been provided [27]. Adherence to these criteria reduces the number of false positives. Evaluation of the models necessary for complex segregation analysis was conducted with the package iBay (version 1.0) [28], an extension of the program MaGGIC [29] written to accommodate binary traits (but not trichotomous traits) in pedigrees with inbreeding. The iBay package [28] was recently used to evaluate the contribution of a major locus to osteochondral diseases in pigs [30], where a more complete outline of the Monte Carlo Markov chains approach is detailed.

The goal of this strategy was to simultaneously estimate the posterior density for a polygenic contribution to binary deafness disease along with the contributions of a putative Mendelian locus. Specifically, for this mixed-inheritance model, the strategy allowed the evaluation of a polygenic variance component, the additive and dominance contributions of a single locus (the parameters -a, d, and a for the putative major locus genotypes AA, AB, and BB, respectively) and the frequency of allele A of the putative major locus (defined as *"q"*). Given our scoring of binary phenotypes, where deaf (both unilateral and bilateral) is 1 and normal is scored as zero, the "B" allele represents the putative disease-enhancing allele. Note also that the iBay software models the unobservable scale of this threshold trait such that the residual variance is fixed at 1.0 (i.e., σ_e_^2 ^= 1).

Creation of the Gibbs sample requires several key assumptions about the behaviour of these unknown parameters. Though a variety of models can be considered, all are some variant of the following: sex, coat texture and coat colour as a fixed effects with a flat (i.e., uniform) prior densities, the polygenic variance component with a flat prior density, as well as flat prior densities for the additive, dominance, and allele frequency parameters. A Gibbs sample of 9,000 was generated, beginning with the creation of 350,000 total samples, a "burn-in" of 50,000 and a sampling rate of every 100-th Gibbs value. This process was repeated two additional times, to create three replicate chains. As outlined above, the post-Gibbs analysis was implemented with the packages boa [20] and coda [21], both part of the R-program [10]. Convergence of the Gibbs sampling process was evaluated visually by sample plots and by contrasting sample means from the first 10% of the sample with the last 50% of the sample [22]. Also as outlined above, from the 9,000 Gibbs samples, the mean, standard deviation, median and the upper and lower limits of a 95% HDR was computed for each of the unknown parameters with hdrcde [24].

## Authors' contributions

TRF participated in design of the study, performed estimation of heritability and complex segregation analysis, and provided review of the manuscript. EJC participated in design of the study, analyzed the phenotypic data, and drafted the manuscript. GMS conceived of the study, participated in design of the study, collected the phenotypic data, and provided review of the manuscript. All authors read and approved the final manuscript.
